# The ASFV CD2v protein inhibits apoptosis by inducing proteasomal degradation of Bim_EL_ via activation of the TPL2-MEK-ERK signaling pathway

**DOI:** 10.1128/jvi.01952-25

**Published:** 2026-02-03

**Authors:** Jianyu Zeng, Xin Zhang, Jingting Zhao, Qiang Li, Zhiyong Xiang, Fengyang Shi, Hua Wang, Wenlian Weng, Qiongqiong Zhou, Peng Gao, Lei Zhou, Xinna Ge, Jun Han, Xin Guo, Yongning Zhang, Hanchun Yang

**Affiliations:** 1 State Key Laboratory of Veterinary Public Health and Safety, College of Veterinary Medicine, China Agricultural University 630101 , Beijing, China; 2 Key Laboratory of Animal Epidemiology of Ministry of Agriculture and Rural Affairs, College of Veterinary Medicine, China Agricultural University 630101 , Beijing, China; Dartmouth College Geisel School of Medicine, Hanover, New Hampshire, USA

**Keywords:** African swine fever virus (ASFV), porcine alveolar macrophages (PAMs), Bim_EL_, TPL2, MEK, ERK, apoptosis

## Abstract

African swine fever virus (ASFV) employs sophisticated regulatory strategies to manipulate host cell apoptosis, a process critical for its pathogenesis and immune evasion; however, the mechanisms underlying this process remain incompletely understood. Here, we report a novel mechanism by which the ASFV-encoded envelope protein CD2v suppresses apoptosis by activating the TPL2 (tumor progression locus 2)-MEK (mitogen-activated protein kinase kinase)-ERK (extracellular signal-regulated kinase) signaling axis, leading to proteasomal degradation of the pro-apoptotic protein Bim_EL_ in primary porcine alveolar macrophages and wild boar lung (WSL) cells. We further demonstrated that ASFV infection triggers ERK1/2-dependent phosphorylation and degradation of Bim_EL_, a process independent of viral replication and mediated by viral structural components. A targeted screen identified CD2v as the key viral protein driving this pathway. Both the purified extracellular domain of CD2v (Asp17−Tyr206) and virion-associated CD2v activated TPL2-MEK-ERK signaling without requiring internalization into cells, resulting in Bim_EL_ downregulation and subsequent suppression of apoptosis. Crucially, CRISPR-Cas9-mediated knockout of CD2v abolished ASFV-induced ERK1/2 activation and consequential Bim_EL_ degradation. Furthermore, we discovered that soluble CD2v released from ASFV-infected cells can activate this signaling axis in uninfected bystander cells, thereby inhibiting apoptosis distantly. This paracrine function, alongside its intrinsic role in directly infected cells, enables CD2v to establish a pro-survival microenvironment conducive to viral propagation. Our findings uncover a multifaceted anti-apoptotic mechanism employed by ASFV, expanding the functional repertoire of CD2v and providing new insights into ASFV pathogenesis with potential therapeutic implications.

**IMPORTANCE:**

This study elucidates a distinct mechanism of apoptosis inhibition by African swine fever virus (ASFV), a pathogen that causes a devastating disease in swine. We identify the ASFV CD2v protein as a key suppressor of cell death that operates by hijacking the host TPL2-MEK-ERK signaling pathway to degrade the pro-apoptotic protein Bim_EL_. Importantly, CD2v mediates this effect not only within infected cells but also, in a soluble form, on surrounding uninfected bystander cells. This dual action helps create a protective, pro-survival cellular environment that facilitates viral spread and persistence. Understanding this novel apoptotic suppression mechanism advances our knowledge of ASFV-host interactions and highlights potential new avenues for therapeutic intervention.

## INTRODUCTION

African swine fever (ASF), caused by the African swine fever virus (ASFV), is a highly lethal disease, with highly pathogenic strains resulting in nearly 100% mortality (1). ASFV is a large, structurally complex double-stranded DNA virus belonging to the genus *Asfvirus* within the family *Asfarviridae*. Its genome, spanning 170–193 kbp, encodes approximately 150–200 proteins, though the functions of most remain poorly understood (1). ASFV primarily infects domestic pigs and Eurasian wild boars, causing severe economic losses to the global swine industry. Acute ASF is typically characterized by high fever, loss of appetite, and lethargy. As the disease progresses, infected pigs may experience anorexia, bloody diarrhea, vomiting, and miscarriage, ultimately leading to death (2). Characteristic pathological changes include vasculitis, skin erythema, pulmonary edema, congestive splenomegaly, hemorrhagic lymphadenitis, and congestion and bleeding points in the kidneys, lungs, and bladder (3). ASFV shows a pronounced tropism for the mononuclear phagocyte system, particularly targeting monocytes and macrophages (4). Microscopic lesions mainly involve the destruction of porcine monocytes and macrophages, lymphocyte depletion, and the death of infiltrating lymphocytes, which have been attributed, at least in part, to the extensive cell apoptosis induced by ASFV infection (5–7), highlighting the critical role of apoptosis in ASFV pathogenicity.

Apoptosis, a genetically programmed cell death process, constitutes a crucial host defense mechanism against intracellular pathogens like viruses. It is mediated through two principal pathways. The extrinsic pathway is initiated by extracellular ligands engaging TNF-family death receptors (8). In contrast, the intrinsic (mitochondrial) pathway is activated by intracellular stress signals and is governed by the Bcl-2 protein family. This family comprises three functional classes: anti-apoptotic proteins (e.g., Bcl-2, Bcl-xL, Mcl-1), which restrain apoptosis under homeostatic conditions by sequestering pro-apoptotic effector proteins (Bax and Bak), pro-apoptotic effector proteins (Bax and Bak), and the pro-apoptotic BH3-only proteins (e.g., Bim, Bid, Puma) (8). Apoptotic stimuli activate BH3-only proteins through diverse transcriptional, translational, and post-translational mechanisms, potentially involving altered subcellular localization (9). Once activated, they promote apoptosis by either indirectly neutralizing anti-apoptotic members or directly engaging and activating Bax/Bak (9). The BH3-only protein Bim exemplifies this dual functionality. It acts as a sensitizer by binding anti-apoptotic proteins via its BH3 domain, thereby liberating sequestered Bax/Bak. Simultaneously, Bim can serve as a direct activator by binding Bax to induce its conformational change, oligomerization, and integration into the mitochondrial outer membrane. This process culminates in mitochondrial outer membrane permeabilization, cytochrome c release, and the irreversible execution of apoptosis (9).

To maintain a stable cellular environment conducive to their replication during early infection, various viruses have evolved mechanisms to inhibit apoptosis (10). ASFV is no exception, having developed complex strategies to suppress apoptosis. Reported ASFV proteins with anti-apoptotic functions include A224L, DP71L, EP153R, A179L, and CD2v (encoded by *EP402R* gene) (11–15). These proteins primarily act by suppressing the pro-apoptotic functions of caspase-3 (CASP3), Bax, and Bak, or by upregulating anti-apoptotic Bcl-2 family proteins, either directly or indirectly. However, the role of pro-apoptotic BH3-only members in ASFV-induced apoptosis and the mechanisms by which ASFV regulates them remain elusive. Many viruses can modulate apoptosis by targeting pro-apoptotic BH3-only members. For instance, Epstein-Barr virus microRNAs target the Bcl-xL/Bcl-2-associated death promoter (BAD), inhibiting apoptosis and maintaining viral latency by downregulating BAD expression (16). Similarly, the viral interferon regulatory factor-1 of human herpesvirus 8 interacts with the BH3 domains of pro-apoptotic proteins such as Bid and Bim, thereby inhibiting their activity (17). In the context of ASFV, research has shown that the pro-apoptotic BH3-only protein Bim, which is normally sequestered by microtubules in healthy cells, redistributes to mitochondria following ASFV infection (18). This suggests that Bim may play a crucial role in ASFV-induced apoptosis. However, the mechanisms by which ASFV regulates Bim and the underlying molecular pathways remain incompletely characterized. In this study, we reveal that ASFV inhibits apoptosis by downregulating Bim through activation of the TPL2 (tumor progression locus 2)-MEK (mitogen-activated protein kinase kinase)-ERK (extracellular signal-regulated kinase) signaling pathway, a process mediated by the ASFV CD2v protein.

## RESULTS

### ASFV infection downregulates the pro-apoptotic protein Bim_EL_ in host cells

**Fig 1:**
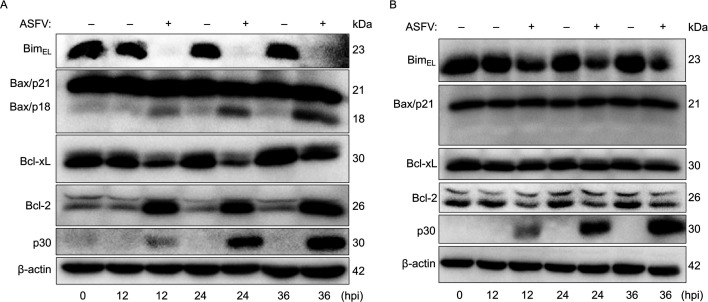
ASFV infection downregulates the pro-apoptotic protein Bim_EL_ in host cells. (**A, B**) Immunoblot analysis of key apoptotic regulators (Bim, Bax, Bcl-xL, Bcl-2), ASFV p30, and β-actin in (**A**) primary PAMs and (**B**) WSL cells that were mock-infected or infected with ASFV (MOI = 0.1) and harvested at the indicated hours post-infection (hpi).

The intrinsic apoptosis pathway is principally governed by the Bcl-2 protein family (8). To investigate whether ASFV infection influences this pathway, we examined the expression of four key Bcl-2 family members with antagonistic roles: the pro-apoptotic proteins Bim and Bax, and the anti-apoptotic proteins Bcl-xL and Bcl-2. The *Bim* gene encodes three major protein isoforms—Bim_EL_ (extra long), Bim_L_ (long), and Bim_S_ (short)—through alternative splicing (19). Among these, Bim_EL_ is typically the most abundant isoform, whereas Bim_L_ and Bim_S_ are frequently undetectable in certain cell types (9). Consistent with this, our western blot analysis confirmed that only Bim_EL_ was expressed at detectable levels in both primary porcine alveolar macrophages (PAMs) and wild boar lung (WSL) cells, with Bim_L_ and Bim_S_ undetectable (data not shown). Further detection revealed that ASFV infection elicited distinct, cell-type-specific alterations in the Bcl-2 family (Fig. 1). Specifically, in PAMs (Fig. 1A), ASFV infection led to specific changes in the expression and activation of Bcl-2 family proteins: (i) Bim_EL_ and Bcl-xL protein levels were significantly downregulated; (ii) Bcl-2 expression was markedly upregulated; and (iii) Bax was activated, as evidenced by cleavage of the full-length protein (Bax/p21) to its active form (Bax/p18), a key indicator of its pro-apoptotic activity (20). In WSL (Fig. 1B), however, a distinct pattern was observed: (i) both Bim_EL_ and Bcl-2 were downregulated; (ii) Bax expression and activation status remained largely unchanged; and (iii) Bcl-xL levels were stable over the course of ASFV infection. Due to the consistent downregulation of the pro-apoptotic Bim_EL_ isoform in both cell types upon ASFV infection, we focused subsequent investigations on this protein.

### Host cell Bim_EL_ levels correlate positively with ASFV-induced apoptosis

**Fig 2:**
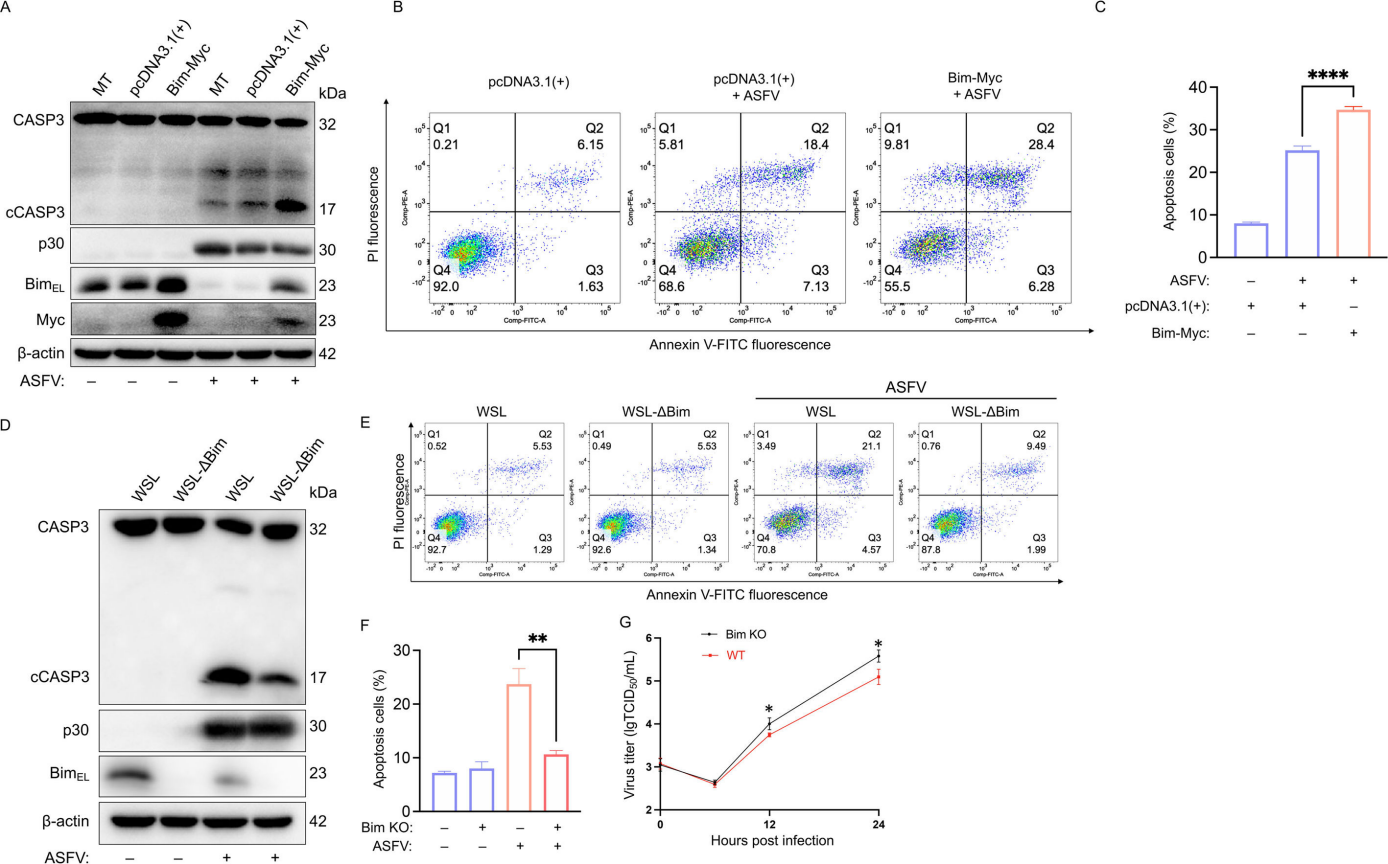
Host cell Bim_EL_ levels are positively correlated with ASFV-induced apoptosis. (**A**) Immunoblot analysis of Myc, Bim_EL_, CASP3, cCASP3, and ASFV p30 in WSL cells that were mock-transfected (MT), transfected with an empty vector (pcDNA3.1(+)), or a Myc-tagged Bim plasmid for 24 h, followed by ASFV infection (MOI = 0.1, 24 h). (**B**) WSL cells, treated as in panel **A**, were analyzed for apoptosis by flow cytometry following Annexin V-FITC/PI staining. (**C**) Quantification of Annexin V-FITC-positive (apoptotic) cells from panel **B**. Data are presented as the mean ± SD of three independent experiments (Student’s *t*-test; ****P < 0.0001). (**D, E**) Immunoblot (**D**) and flow cytometry (**E**) analysis of apoptosis in wild-type and Bim knockout WSL (WSL-ΔBim) cells following ASFV infection (MOI = 0.1, 36 h). (**F**) Quantification of apoptotic cells from (**E**). Data are presented as the mean ± SD of three independent experiments (Student’s *t*-test; **P < 0.01). (**G**) Viral growth kinetics in wild-type and WSL-ΔBim cells infected with ASFV (MOI = 0.1). Total viral titers from combined intracellular and extracellular fractions were determined by TCID₅₀ assay at indicated time points. Data are presented as the mean ± SD of three independent experiments (Student’s *t*-test; *P < 0.05).

Bim_EL_ is a pro-apoptotic protein whose activity is regulated through sequestration by the microtubular cytoskeleton or by forming inactive heterodimers with anti-apoptotic Bcl-2 family members on mitochondria (9). To investigate the functional role of Bim_EL_ in ASFV-induced apoptosis, we employed both gain- and loss-of-function approaches. Overexpression of Bim in WSL cells significantly enhanced the yield of cleaved caspase-3 (cCASP3)—a key executioner molecule of apoptosis—upon ASFV infection (Fig. 2A). This pro-apoptotic effect was further validated by a corresponding increase in apoptotic cells quantified via Annexin V/PI staining-based flow cytometry (Fig. 2B and C). Conversely, transfection with two specific siRNAs (si*Bim*-1/2), which efficiently knocked down Bim_EL_ expression (Fig. S1A), markedly reduced cCASP3 production in ASFV-infected WSL cells (Fig. S1B). The attenuation of apoptosis following Bim knockdown was confirmed by flow cytometric analysis (Fig. S1C and D). To further substantiate these findings, we generated a Bim knockout (Bim KO) WSL cell line (WSL-ΔBim). Consistent with the knockdown results, ASFV-induced apoptosis was significantly attenuated in the KO cells (Fig. 2D through F). Furthermore, ASFV replication was significantly enhanced in Bim KO cells compared to wild-type controls (Fig. 2G). Collectively, these data demonstrate that Bim_EL_ expression not only promotes ASFV-induced apoptosis but also restricts viral replication, underscoring its dual role in the host response to infection.

### ASFV triggers proteasomal degradation of Bim_EL_ via ERK1/2-dependent phosphorylation

**Fig 3:**
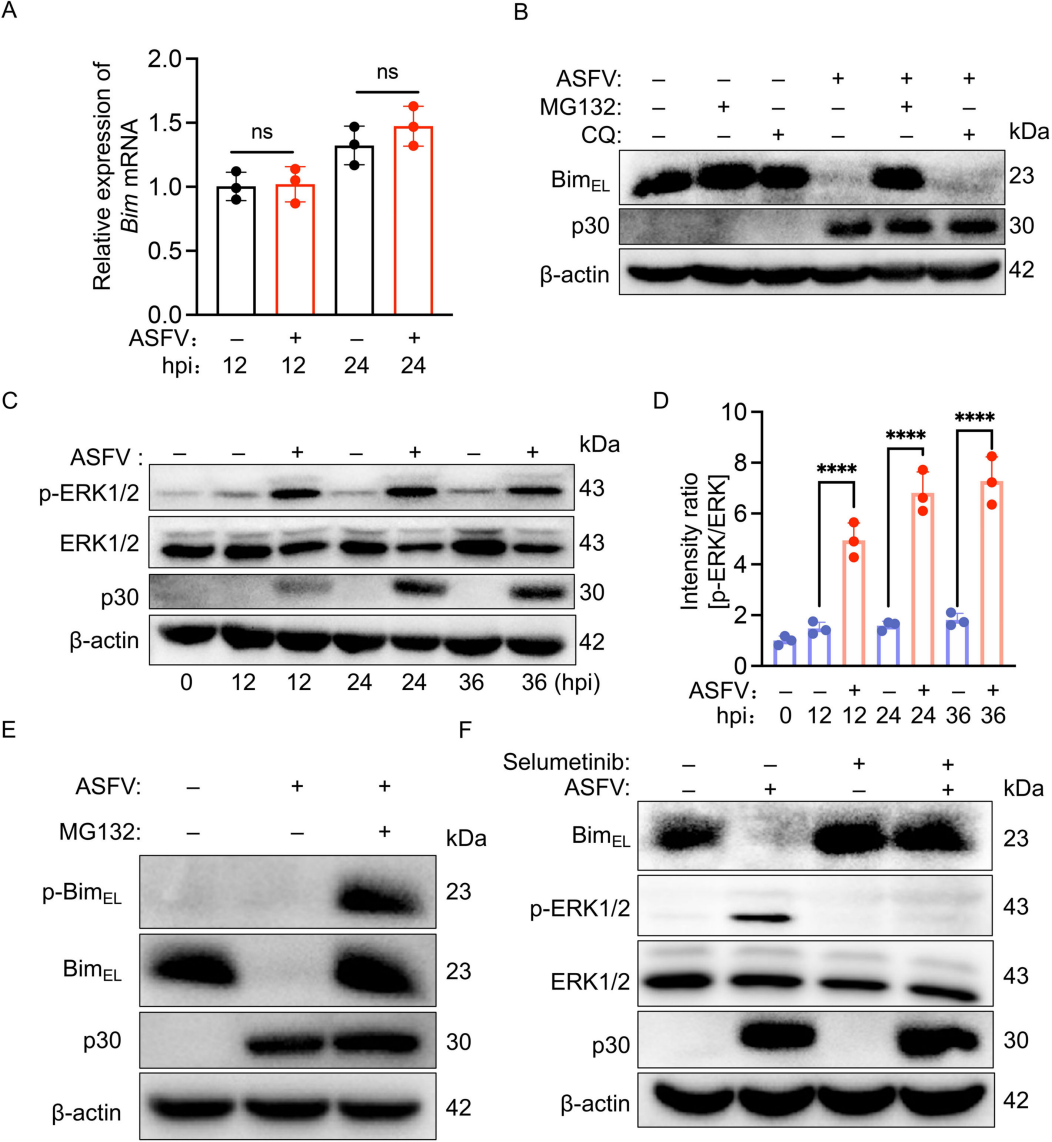
ASFV drives proteasomal degradation of Bim_EL_ through ERK1/2-dependent phosphorylation. (**A**) qPCR analysis of *Bim* mRNA levels in mock- or ASFV-infected PAMs (MOI = 0.1) at the indicated time points. Data, normalized to β-actin, show the mean ± SD of three independent experiments (Student’s *t*-test; ns, no significance). (**B**) Immunoblotting analysis of Bim in mock- or ASFV-infected PAMs (MOI = 0.1, 24 h) treated with the proteasome inhibitor MG132 (5 μM) or the lysosome inhibitor CQ (5 μM). The cell lysates were probed for Bim_EL_, p30, and β-actin. (**C**) Immunoblot analysis of ERK1/2 activation in PAMs over time following ASFV infection (MOI = 0.1). Cells were harvested at the indicated time points post-infection, and lysates were probed for p-ERK1/2, ERK1/2, p30, and β-actin. (**D**) Densitometric quantification of the p-ERK/ERK ratio from (**C**). Data are presented as mean ± SD of three independent experiments (Student’s *t*-test; ****P < 0.0001). (**E**) Immunoblot analysis of Bim_EL_ phosphorylation in PAMs infected with ASFV (MOI = 0.1, 24 h) and treated with or without 5 μM MG132. Lysates were probed with an antibody specific for Bim_EL_ phosphorylated at serine 69 (p-Bim_EL_), alongside antibodies for total Bim_EL_, p30, and β-actin. (**F**) Immunoblot analysis of the role of the ERK1/2 pathway in ASFV-induced Bim_EL_ degradation. PAMs were mock- or infected with ASFV (MOI = 0.1) for 24 h in the presence or absence of 0.1 μM selumetinib. Lysates were probed for Bim_EL_, p-ERK1/2, ERK1/2, p30, and β-actin.

Current research indicates that various viruses modulate Bim expression through distinct mechanisms, with some influencing transcription and others affecting post-translational processes (21, 22). To investigate how ASFV infection leads to Bim_EL_ downregulation, we infected PAMs with ASFV and assessed Bim transcription using quantitative real-time PCR (qPCR). Our data revealed that ASFV infection did not significantly alter Bim transcription at 12 or 24 hpi (Fig. 3A), suggesting that Bim_EL_ downregulation may result from protein degradation. Since lysosomal and proteasomal pathways are the two main routes of intracellular protein degradation, we treated ASFV-infected PAMs with the lysosome inhibitor chloroquine (CQ) or the proteasome inhibitor MG132. Immunoblotting results showed that CQ had no significant effect on Bim_EL_ levels, whereas MG132 prevented Bim_EL_ downregulation (Fig. 3B). These findings indicate that ASFV infection downregulates Bim_EL_ primarily through the proteasome pathway. Multiple studies have demonstrated that phosphorylated ERK1/2 (p-ERK1/2) promotes the proteasomal degradation of Bim_EL_ by phosphorylating it at serine 69 (23). Given that ERK1/2 phosphorylation is known to be upregulated in PAMs upon infection with ASFV (24), we sought to investigate this signaling axis. Consistent with the literature, our immunoblotting analysis confirmed a significant increase in p-ERK1/2 levels following ASFV infection (Fig. 3C and D). To determine whether this ASFV-induced ERK1/2 activation leads to the phosphorylation of Bim_EL_ at serine 69, we probed cell lysates from ASFV-infected PAMs, treated with or without the proteasome inhibitor MG132, using a monoclonal antibody specific for Bim_EL_ phosphorylated at serine 69. The results demonstrate that ASFV infection indeed induces Bim_EL_ phosphorylation at serine 69 (Fig. 3E). Furthermore, to determine whether ERK1/2 is responsible for the downregulation of Bim_EL_, we treated ASFV-infected PAMs with Selumetinib, an inhibitor of MEK1/2 (the upstream kinase of ERK1/2) (25), using DMSO-treated cells as a solvent control. In Selumetinib-treated PAMs, both ASFV-induced ERK1/2 activation and Bim_EL_ downregulation were abolished (Fig. 3F). Collectively, these findings indicate that ASFV infection activates the ERK1/2 signaling to promote ERK1/2 phosphorylation and its subsequent proteasomal degradation.

### Inhibition of the ERK1/2 pathway potentiates ASFV-induced apoptosis

**Fig 4:**
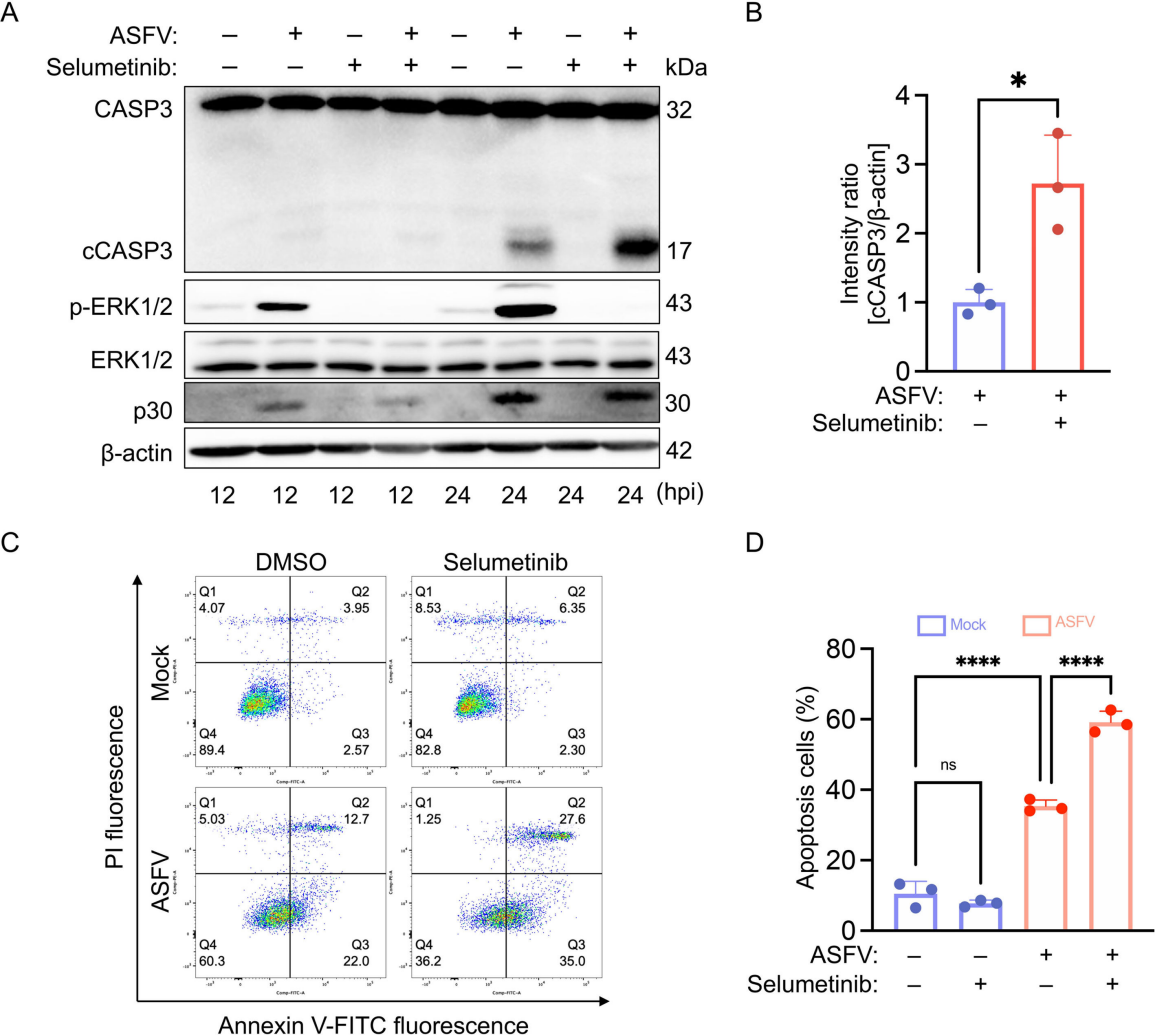
Inhibition of the ERK1/2 signaling pathway enhances ASFV-induced apoptosis. (**A**) Immunoblotting analysis of PAMs mock-infected or infected with ASFV (MOI = 0.1) and treated with 0.1 μM selumetinib. Cells were harvested at the indicated time points and probed for CASP3, cCASP3, p-ERK1/2, ERK1/2, p30, and β-actin. (**B**) Densitometric quantification of the p-ERK/ERK ratio from panel **A**. Data are presented as mean ± SD of three independent experiments (Student’s *t*-test; *P < 0.05). (**C**) Flow cytometric analysis of apoptosis in PAMs, treated and infected as in panel **A** for 24 h, following Annexin V-FITC/PI staining. (**D**) Quantification of Annexin V-FITC-positive (apoptotic) cells from panel **C**. Data are presented as the mean ± SD of three independent experiments (Student’s *t*-test; ****P < 0.0001; ns, no significance).

Previous studies have demonstrated that the ERK1/2 signaling pathway can play a dual role in either promoting or inhibiting apoptosis, depending on the specific stimulus conditions (26). To explore the role of ERK1/2 in ASFV-induced apoptosis, PAMs infected with ASFV were simultaneously treated with Selumetinib, while DMSO-treated cells served as a solvent control. Selumetinib significantly inhibited ERK1/2 phosphorylation and increased cCASP3 levels compared to the control (Fig. 4A and B). Additionally, Annexin V/PI double-staining by flow cytometry revealed that Selumetinib treatment markedly enhanced ASFV-induced apoptosis (Fig. 4C and D). These results indicate that ASFV-induced apoptosis is markedly enhanced by inhibition of the ERK1/2 signaling pathway.

### Overactivation of the ERK1/2 signaling pathway inhibits ASFV replication

**Fig 5:**
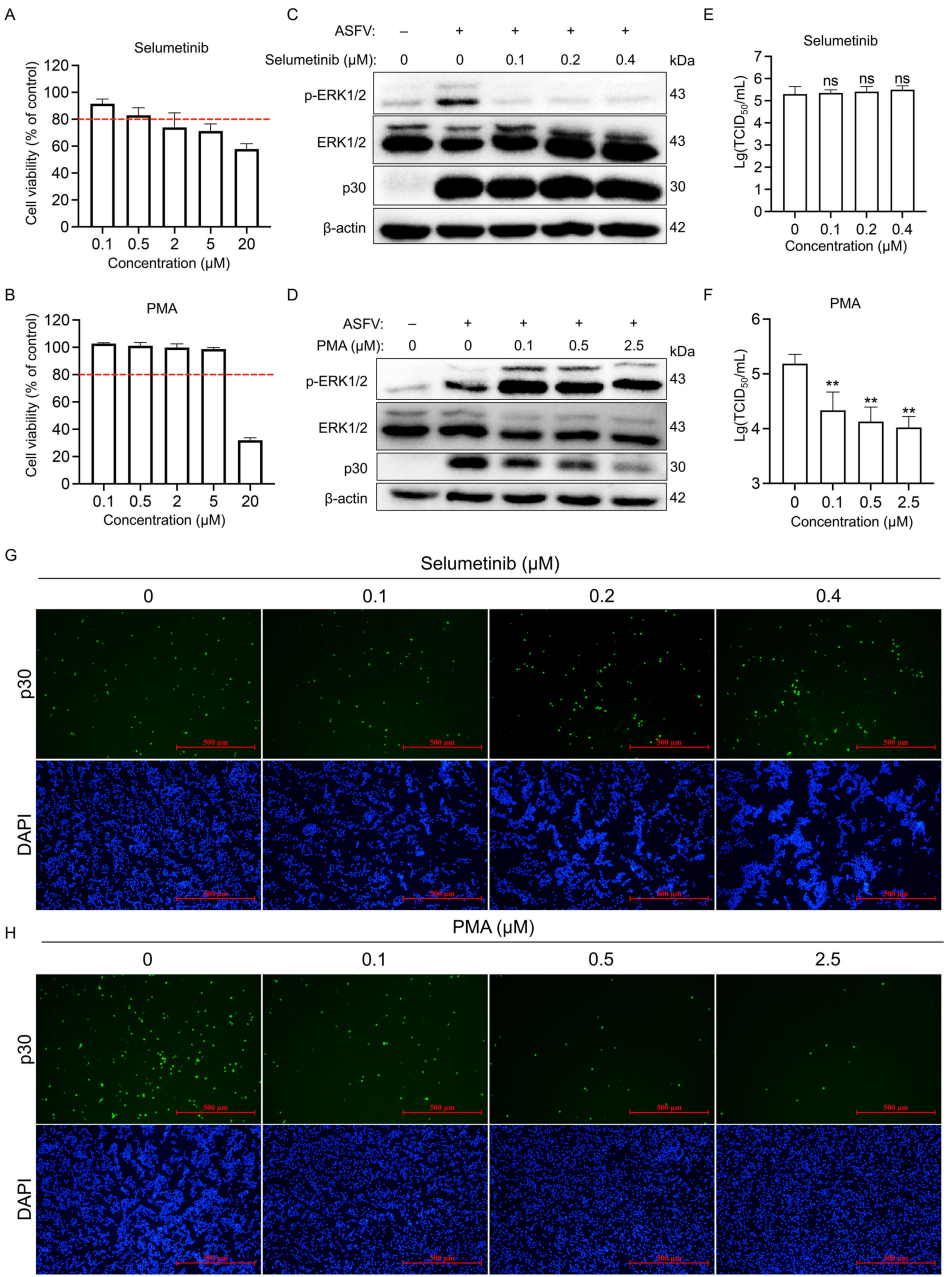
Analysis of the impact of ERK1/2 signaling pathway disruption on ASFV replication. (**A, B**) Viability of PAMs treated with various concentrations of Selumetinib (**A**) or PMA (**B**), assessed by CCK-8 assay. The red dotted line indicates 80% cell viability. (**C, D**) Immunoblotting analysis of PAMs, either mock-infected or infected with ASFV (MOI = 0.1), concurrently treated with Selumetinib (**C**) or PMA (**D**) for 24 h. Cell lysates were subjected to immunoblotting for p-ERK1/2, ERK1/2, p30, and β-actin. (**E, F**) Virus titers in PAMs under the same infection and treatment conditions as in panels **C and D**, as measured by the TCID_50_ assay. Data are presented as the mean ± SD of three independent experiments (Student’s *t*-test; **P < 0.01; ns, no significance). (**G, H**) IFA analysis of infected and treated PAMs as in panels **E and F**. Fixed cells were immunostained with an anti-p30 primary antibody and an Alexa Fluor 488-conjugated secondary antibody, followed by DAPI counterstaining, and visualized by fluorescence microscopy.

Given the established role of the ERK1/2 signaling pathway in regulating multiple viral life cycles and its potential as a target for antiviral intervention (27), we sought to investigate whether it also influences ASFV replication. To modulate ERK1/2 activity, we used the MEK inhibitor Selumetinib for suppression and phorbol myristate acetate (PMA)—a diacylglycerol analog that activates protein kinase C to initiate the RAF-MEK-ERK cascade—for activation (28). First, we determined the non-cytotoxic concentration ranges for both compounds through cell viability assays, applying an 80% viability threshold. The safe concentrations were defined as >0.5 µM for Selumetinib and >5 µM for PMA in PAMs (Fig. 5A and B). Immunoblot analysis confirmed the efficacy of both modulators: Selumetinib at 0.1, 0.2, and 0.4 μM effectively suppressed ERK1/2 phosphorylation, whereas PMA at 0.1, 0.5, and 2.5 μM strongly induced it (Fig. 5C and D). We then evaluated the effect of ERK1/2 modulation on ASFV replication by quantifying virus titers and performing an indirect immunofluorescence assay (IFA) using an antibody against the ASFV p30 protein. Notably, Selumetinib-mediated inhibition of ERK1/2 phosphorylation did not significantly alter ASFV replication (Fig. 5E), a result corroborated by IFA (Fig. 5G). In contrast, PMA-induced hyperactivation of ERK1/2 suppressed ASFV replication in a concentration-dependent manner, as evidenced by reduced viral titers (Fig. 5F) and IFA signals (Fig. 5H). The divergent effects of inhibition versus activation may stem from the functional versatility of the ERK1/2 pathway, which regulates both apoptosis and pro-inflammatory immune responses (29). We propose that Selumetinib neutralizes these opposing functions, resulting in no net change in ASFV replication. Collectively, our data demonstrate that ERK1/2 overactivation, but not its inhibition, significantly curtails ASFV replication.

### ASFV activates the TPL2-MEK-ERK signaling axis independently of viral replication

**Fig 6:**
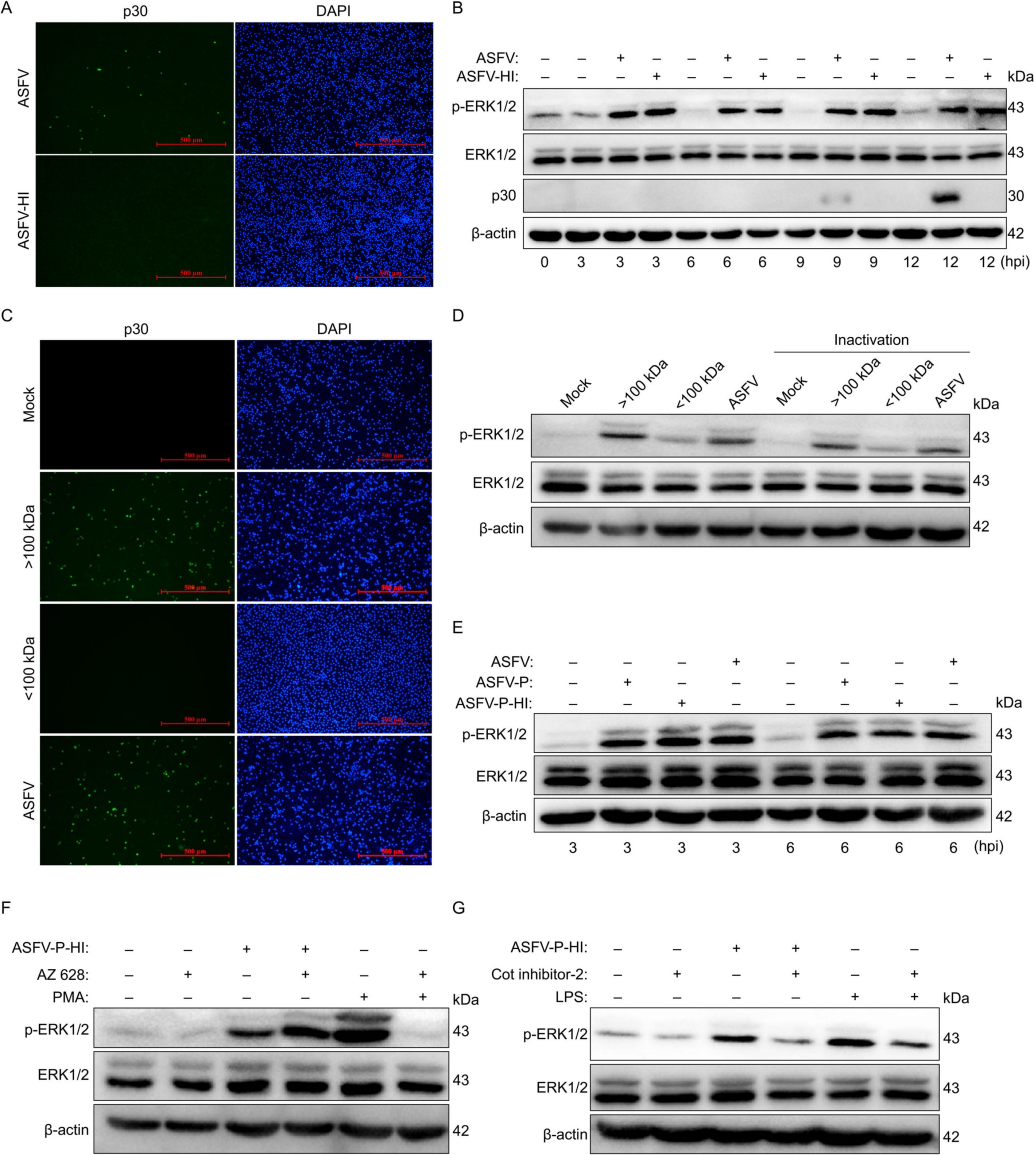
ASFV activates the TPL2-MEK-ERK signaling axis independently of viral replication. (**A**) IFA confirming the efficacy of ASFV inactivation. PAMs were inoculated with live or ASFV-HI (MOI = 0.1) for 12 h. The cells were then fixed, immunostained with an anti-p30 mAb primary antibody and an Alexa Fluor 488-conjugated secondary antibody, and counterstained with DAPI before visualization by fluorescence microscopy. (**B**) Immunoblotting analysis of ERK1/2 activation by live versus inactivated virus. PAMs were mock-infected or inoculated with live ASFV or ASFV-HI (MOI = 0.1) and harvested at the indicated time points. Cell lysates were subjected to immunoblotting for p-ERK1/2, ERK1/2, p30, and β-actin. (**C**) IFA assessing the infectivity of size-fractionated ASFV. PAMs were mock-infected or inoculated with unfractionated ASFV, or the >100 kDa or <100 kDa ultrafiltration fractions for 24 h. The cells were fixed and analyzed as in panel **A**. (**D**) Immunoblotting analysis of ERK1/2 activation by size-fractionated ASFV, live, and heat-inactivated ASFV. PAMs were mock-infected or inoculated with the >100 kDa or <100 kDa fractions for 3 h, and cell lysates were immunoblotted for the indicated proteins. (**E**) Immunoblotting analysis of ERK1/2 activation by different viral preparations. PAMs were either mock-infected or inoculated with unpurified ASFV, ASFV-P, or ASFV-P-HI and collected at indicated time points for immunoblotting. (**F, G**) The role of the TPL2-MEK-ERK axis in ASFV-induced apoptosis was probed using specific inhibitors. PAMs were pretreated for 3 h with either AZ 628 (RAF inhibitor, 5 μM), or Cot inhibitor-2 (TPL2 inhibitor, 20 μM), followed by stimulation for 3 h with ASFV-P-HI, PMA (RAF activator, 2.5 μM), or LPS (TPL2 activator, 1 μg/mL). Phosphorylation of ERK1/2 was subsequently analyzed via immunoblotting.

Previous studies have demonstrated that various viruses activate the ERK1/2 signaling pathway through distinct mechanisms (30). To determine how ASFV triggers this pathway, we first assessed whether viral replication is required for ERK1/2 activation by using heat-inactivated ASFV (ASFV-HI; 70°C for 40 min). Initial IFA confirmed the complete loss of infectivity (Fig. 6A). Subsequent immunoblotting revealed that, similar to the live virus, ASFV-HI retained the ability to activate ERK1/2 (Fig. 6B). To exclude potential confounding effects from small molecules (e.g., cytokines) in ASFV-infected supernatants, we used 100 kDa ultrafiltration tubes to separate virions from smaller components. IFA confirmed that only the >100 kDa fraction remained infectious (Fig. 6C), validating the separation efficiency. Immunoblotting revealed that the >100 kDa fraction was the primary contributor to ERK1/2 activation, although the <100 kDa fraction exhibited weak activation (Fig. 6D). To further verify that the virion itself is primarily responsible for ERK1/2 activation, we purified ASFV virions using density gradient centrifugation. The purified ASFV (ASFV-P), purified and heat-inactivated ASFV (ASFV-P-HI), and unpurified ASFV were inoculated into PAMs to assess their ability to activate ERK1/2. Immunoblotting showed that all three preparations exhibited comparable ERK1/2 activation (Fig. 6E). These results indicate that ERK1/2 activation is triggered either by the ASFV virion itself or a specific viral particle component, independent of viral replication.

In addition, the mitogen-activated protein kinase (MAPK) pathway represents a fundamental signaling cascade in eukaryotic cells, characterized by a three-tiered activation mechanism: MAPK kinase kinases (MAP3Ks) initiate the cascade by phosphorylating MAPK kinases (MAP2Ks), which then activate MAPKs (31). In mammalian cells, the rapidly accelerated fibrosarcoma (RAF) protein kinase—a classical MAP3K responsive to receptor tyrosine kinase (RTK) signaling—typically mediates MEK (MAP2K)-ERK (MAPK) activation (32). However, in primary PAMs, TPL2, a myeloid-enriched MAP3K that responds to immune receptors (TLRs, TNFR1, and IL-1R), may represent the predominant ERK1/2 activator (33). To identify which MAP3K is responsible for ASFV-induced ERK activation, we employed specific pharmacological modulators: AZ 628 (RAF inhibitor) and Cot inhibitor-2 (TPL2 inhibitor), with PMA (RAF activator) and lipopolysaccharide (LPS) (TPL2 activator) serving as positive controls (28, 34–36). Notably, while AZ 628 blocked PMA-induced ERK1/2 phosphorylation, it failed to inhibit ERK1/2 activated by ASFV-P-HI (Fig. 6F). In contrast, Cot inhibitor-2 effectively suppressed ERK1/2 phosphorylation induced by both LPS and ASFV-P-HI (Fig. 6G). These results indicate that ASFV-P-HI activates ERK1/2 through TPL2 rather than RAF. Taken together, these findings further demonstrate that viral structural components alone can activate the TPL2-MEK-ERK signaling axis independently of replicative competence.

### ASFV CD2v protein drives Bim_EL_ degradation and consequent apoptosis suppression

**Fig 7:**
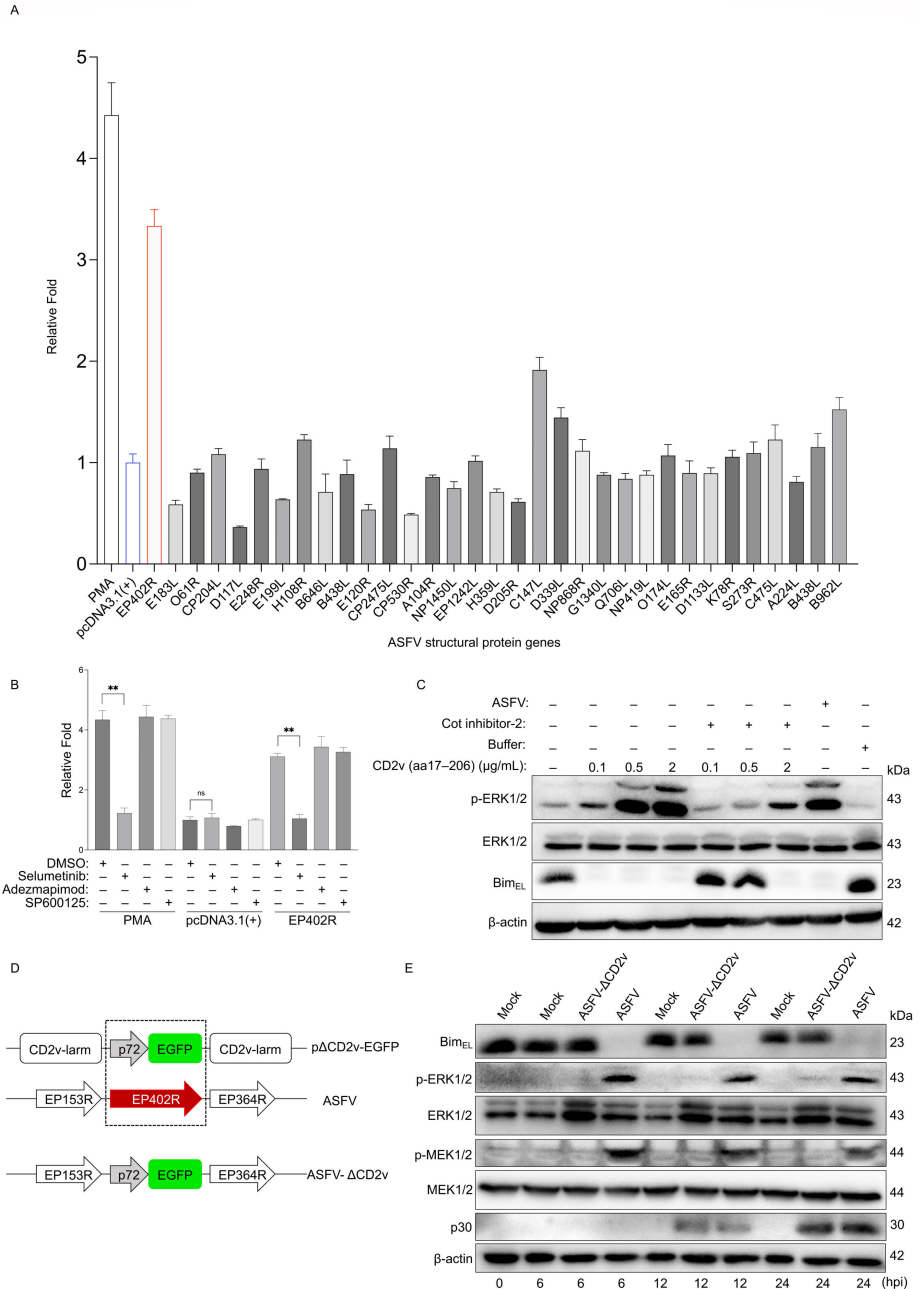
The ASFV CD2v protein mediates ERK1/2 activation and subsequent Bim_EL_ degradation. (**A**) AP-1 transcriptional activity was assessed in WSL cells transfected for 36 h with an empty vector (pcDNA3.1(+)) or plasmids expressing individual ASFV structural proteins, using a dual-luciferase reporter assay. Results are presented relative to the empty vector control, with 2 μM PMA as a positive control. (**B**) WSL cells were transfected with the empty vector or an *EP402R* (CD2v)-expressing plasmid. At 12 h post-transfection, cells were treated with the MEK inhibitor Selumetinib (2 μM), the p38 MAPK inhibitor Adezmapimod (5 μM), or the JNK inhibitor SP600125 (20 μM) for 24 h before assessing AP-1 activity. (**C**) PAMs pretreated with Cot inhibitor-2, at a final concentration of 20 μM, or DMSO for 3 h were stimulated with the purified CD2v protein (Asp17–Tyr206) (at a final concentration of 0.1, 0.5, or 2 μg/mL) for 30 min, and detected by immunoblotting using antibodies against p-ERK1/2, ERK1/2, and β-actin. PAMs stimulated with buffer or ASFV (MOI = 0.1) for 30 min were set as the negative or positive control, respectively. (**D**) Schematic of the ASFV-ΔCD2v mutant, where the *EP402R* gene was replaced with an EGFP cassette under the control of the ASFV p72 promoter. (**E**) Immunoblot analysis of PAMs mock-infected or infected with wild-type ASFV or ASFV-ΔCD2v (MOI = 0.1) for the indicated time points. Cell lysates were probed for p-ERK1/2, ERK1/2, Bim, p30, and β-actin.

The activator protein 1 (AP-1) transcription factor is a well-characterized downstream target phosphorylated and activated by ERK1/2 (37). To enable high-throughput, quantitative detection of ERK1/2 activation, we employed an AP-1–luciferase reporter assay. This assay provides a rapid and specific readout for ERK1/2–AP-1 axis activation, offering significant advantages over monitoring Bim_EL_ downregulation, which is slower and can be influenced by ERK1/2-independent mechanisms. Based on our finding that viral structural components activate ERK1/2 (Fig. 6), we screened 33 ASFV structural protein-encoding genes with this assay, using PMA and an empty vector pcDNA3.1(+) as positive and negative controls, respectively. This screen identified CD2v, encoded by *EP402R*, as the most potent activator of AP-1 transcriptional activity (Fig. 7A). Since the MAPK family members ERK1/2, c-Jun N-terminal kinase (JNK), and p38 can all activate AP-1 transcription (38), we employed selective pharmacological inhibitors to delineate the pathway responsible for CD2v-mediated activation (24). Prior to formal experiments, we determined non-cytotoxic concentrations of each inhibitor (maintaining >80% cell viability in WSL cells) to be >2 µM for Selumetinib (MEK1/2 inhibitor), > 5 μM for Adezmapimod (p38 inhibitor), and > 20 μM for SP600125 (JNK inhibitor) (Fig. S2). At the determined non-cytotoxic concentrations, only the MEK1/2 inhibitor Selumetinib effectively inhibited CD2v-mediated AP-1 transcriptional activity (Fig. 7B), suggesting that CD2v activates AP-1 primarily through the ERK1/2 pathway and not via p38 or JNK. To investigate whether CD2v acts through the TPL2-MEK-ERK signaling axis, we pretreated PAMs with the TPL2 inhibitor Cot inhibitor-2, followed by stimulation with purified mammalian-expressed CD2v extracellular domain (Asp17–Tyr206). Immunoblotting demonstrated that recombinant CD2v dose-dependently activated ERK1/2 and downregulated Bim_EL_, effects that were partially attenuated by Cot inhibitor-2, confirming CD2v’s engagement of this upstream axis (Fig. 7C). We next assessed whether CD2v internalization is required for this signaling. Pretreatment of PAMs with 50 μM chlorpromazine (a clathrin-mediated endocytosis inhibitor) or incubation at 4°C (to block energy-dependent internalization) did not affect CD2v-induced ERK1/2 phosphorylation or Bim_EL_ downregulation (Fig. S3), demonstrating that internalization is not necessary for pathway activation. To definitively link CD2v to the inhibition of apoptosis, we constructed a CD2v-knockout ASFV mutant (ASFV-ΔCD2v) using the strategy in Fig. 7D. PCR identification with *EP402R*-specific primers confirmed gene deletion in ASFV-ΔCD2v, while *B646L*-specific primers produced amplicons from both viruses (Fig. S4A). The successful construction of ASFV-ΔCD2v was verified through fluorescence microscopy detecting EGFP expression in infected PAMs (Fig. S4B) and confirmed by sequencing (data not shown). The mutant exhibited growth kinetics in PAMs comparable to the parental virus (Fig. S4C). Crucially, immunoblot analysis revealed that ASFV-ΔCD2v nearly lost the ability to induce both ERK1/2 activation and Bim_EL_ degradation in PAMs (Fig. 7E) and WSL cells (Fig. S5). Collectively, these results establish that the ASFV CD2v protein is both necessary and sufficient for activating the TPL2-MEK-ERK signaling axis and subsequent Bim_EL_ downregulation.

## DISCUSSION

**Fig 8:**
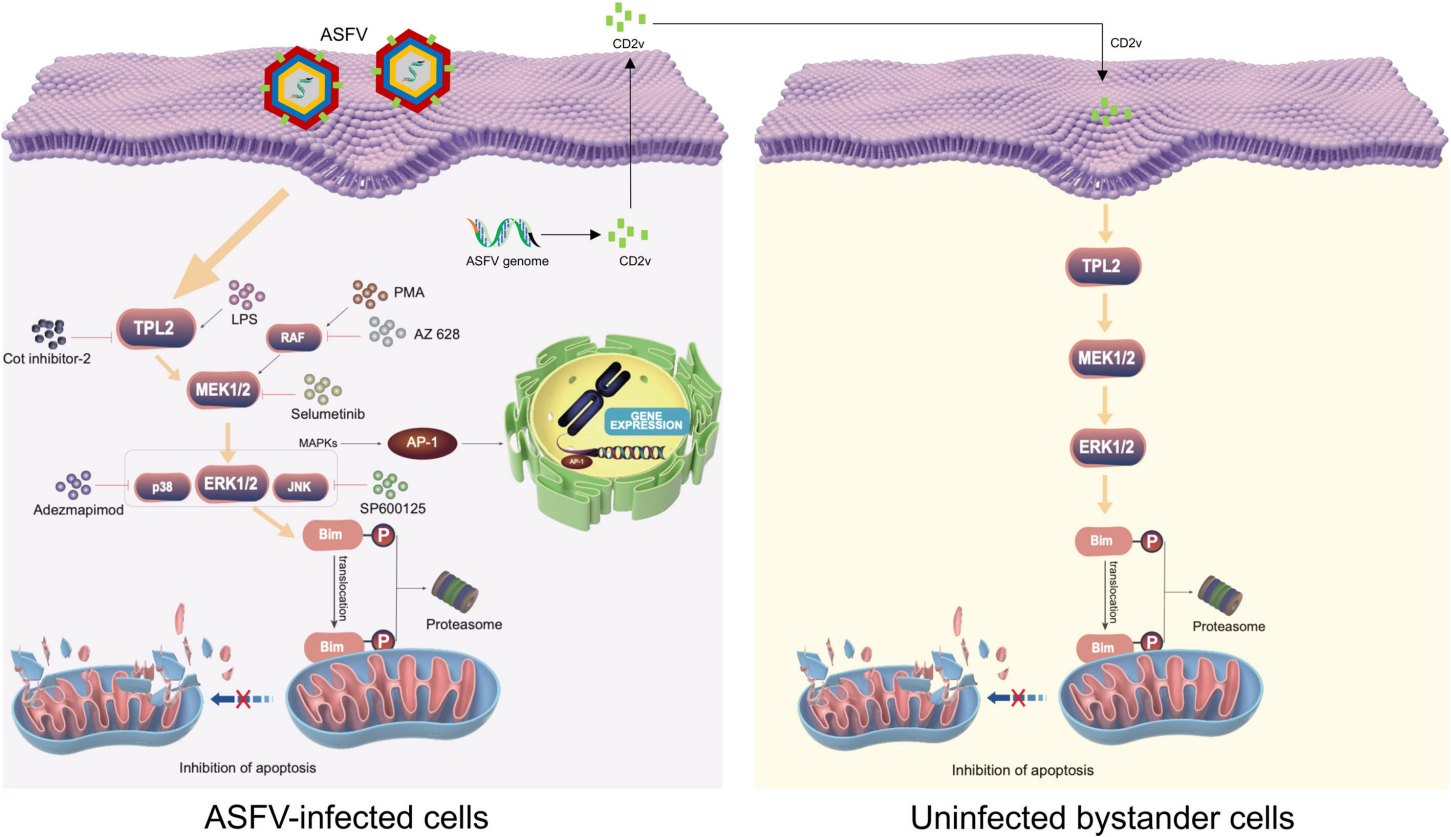
A proposed model for ASFV CD2v-mediated apoptosis inhibition in host cells. The CD2v protein, present in both virion-associated and soluble forms (released from infected cells), suppresses apoptosis in ASFV-infected and uninfected bystander cells, respectively, by activating the TPL2-MEK-ERK signaling axis. This activation leads to phosphorylation of Bim_EL_ specifically by ERK1/2 (not JNK or p38), targeting the pro-apoptotic protein Bim_EL_ for proteasomal degradation and thereby inhibiting apoptosis. The pharmacological modulators used are specified in the figure.

Apoptosis plays a pivotal role in ASFV pathogenesis, significantly contributing to its hallmark clinical manifestations—such as pyrexia, hemorrhagic lesions, and lymphadenopathy—in infected swine (2). Following host infection, ASFV exhibits a dual regulatory strategy toward apoptosis: it triggers apoptosis to facilitate viral dissemination and cause extensive tissue damage, resulting in massive cellular depletion and lymphocytopenia in affected organs (7). Paradoxically, ASFV also employs apoptosis suppression strategies to establish persistent infection and enhance viral replication within host cells (11). Current research reveals that ASFV dynamically modulates apoptotic pathways through sophisticated and diverse regulatory mechanisms, with distinct viral proteins functioning either as apoptosis inhibitors or inducers during different stages of infection. Currently identified ASFV-encoded apoptosis inhibitors include A224L, DP71L, EP153R, A179L, and CD2v (11–15), while apoptosis inducers identified comprise E183L (p54), E199L, MGF360-16R, and CD2v (18, 20, 39, 40). Notably, CD2v exhibits conflicting apoptotic regulation: it inhibits apoptosis in PAMs via JAK2-STAT3 activation (15), yet promotes apoptosis in peripheral blood mononuclear cells (PBMCs) and macrophages through nuclear factor kappa-B (NF-κB)-mediated IFN signaling (40). This functional dichotomy may reflect strain-dependent differences in CD2v activity. Previous studies demonstrated that the pro-apoptotic protein Bim translocates to mitochondria in ASFV-infected cells (18). Amino acid sequence analysis revealed similarity between the DLC8-binding motif of Bim and the dynein-binding motif of ASFV p54 protein, potentially explaining p54’s pro-apoptotic function (41). These findings highlight Bim’s critical role in ASFV-regulated apoptosis, though the underlying mechanisms remained unclear. In the present study, we demonstrate that ASFV CD2v—in both its virion-associated and free soluble forms—suppresses apoptosis in ASFV-infected cells and uninfected bystander cells, respectively, through a mechanism involving activation of the TPL2-MEK-ERK signaling axis, which induces proteasomal degradation of the pro-apoptotic protein Bim_EL_. This mechanism helps reconcile CD2v’s previously observed anti-apoptotic function. A proposed model summarizing CD2v-mediated Bim_EL_ degradation and the resulting suppression of apoptosis is presented in Fig. 8.

Given that the NF-κB and ERK1/2 pathways often share upstream signals, such as TNFR1, IL1R, or TLRs, we investigated their interplay (32). These receptors trigger downstream activation of the IKK complex (comprising IKKα, IKKβ, and IKKγ/NEMO), which mediates the degradation of IκB and the TPL2 inhibitory protein p105, thereby activating NF-κB and TPL2 (32). Notably, we found that ASFV induces ERK1/2 activation via the TPL2-MEK-ERK axis rather than the canonical RAF-MEK-ERK cascade. TPL2, a MAP3K highly expressed in myeloid cells and fibroblasts, plays key roles in inflammation and angiogenesis (33). Intriguingly, TPL2—but not RAF—shares upstream signals, suggesting that CD2v may stimulate TNFR1/IL1R/TLRs-IKK signaling to concurrently engage both NF-κB and ERK1/2 signaling in macrophages. The pro- or anti-apoptotic effects of CD2v may thus hinge on the balance between these pathways. Contrasting with our findings, a prior study reported that CD2v suppresses ERK1/2 activation in immortalized PAMs 3D4/21 (42). This discrepancy likely arises from differences in cell types: our experiments used primary PAMs from SPF pigs and immortalized WSL cells from wild boars, whereas 3D4/21 cells, derived from domestic pigs, poorly support ASFV HN09 replication. Of note, we attempted to identify direct host interactors of CD2v upstream of TPL2 using co-immunoprecipitation coupled with mass spectrometry. This screen yielded several candidate host interactors—including VDAC2, vimentin, cofilin-1, and HSPA8—but did not detect any previously reported upstream regulators of TPL2 (33). This result suggests that the relevant interactor(s) may be transient, low-affinity, or indirect, and therefore not readily captured by our current experimental approach. This gap underscores the need for future studies employing alternative approaches to identify the potential host factor(s) involved, which is essential for a more detailed understanding of how ASFV exploits this pathway to inhibit apoptosis. Nevertheless, our current results definitively demonstrate that CD2v is both necessary and sufficient to activate the TPL2-MEK-ERK signaling axis, leading to the proteasomal degradation of Bim_EL_ and consequently inhibiting apoptosis in both ASFV-infected target cells and uninfected bystander cells. Activation by the extracellular domain of CD2v occurs independently of internalization (Fig. S3). Together, these findings support a model in which the extracellular domain of CD2v engages one or more as-yet-unidentified host cell surface molecules—potentially in a multivalent manner—to trigger an intracellular signaling cascade that culminates in TPL2 activation. Identifying such upstream host factor(s) remains a primary focus of our ongoing research and will be essential for fully elucidating the role of CD2v in ASFV pathogenesis and for developing targeted antiviral interventions.

Our study demonstrates that the major Bcl-2 protein family members we analyzed underwent distinct, cell-type-specific reprogramming during ASFV infection (Fig. 1). Notably, the pro-apoptotic BH3-only protein Bim—a key sentinel of the mitochondrial apoptotic pathway (19)—was consistently downregulated in both ASFV-infected PAMs and WSL cells. This downregulation is strategically significant, as Bim promotes apoptosis by directly neutralizing anti-apoptotic proteins (e.g., Bcl-2, Bcl-xL, Mcl-1) or activating the pro-apoptotic effectors Bax/Bak, events that converge on mitochondrial outer membrane permeabilization to trigger cell apoptosis (9). Previous studies revealed that ASFV infection induces Bim redistribution to mitochondria, implicating its involvement in ASFV-induced apoptosis (18). Here, we definitively established Bim’s critical role in this process: its overexpression enhanced ASFV-triggered apoptosis, whereas both siRNA-mediated knockdown and CRISPR-Cas9-mediated knockout significantly attenuated cell apoptosis. Bim expression and activity are tightly regulated at transcriptional, translational, and post-translational levels (9). For instance, ERK1/2—a member of the MAPK family—phosphorylates Bim_EL_ at serine 69, targeting it for proteasomal degradation (23). Several viruses exploit this regulatory mechanism to modulate apoptosis: Epstein-Barr virus, Varicella-Zoster virus, and Pseudorabies virus downregulate Bim_EL_ via ERK1/2 activation to suppress apoptosis (21, 43, 44), whereas Hepatitis C virus upregulates Bim transcription via the ROS/JNK pathway to promote apoptosis (22). Our data show that ASFV infection downregulates Bim_EL_ protein post-transcriptionally, as evidenced by unchanged mRNA levels. Mechanistically, we demonstrate that the ASFV CD2v protein activates the TPL2-MEK-ERK pathway to promote Bim_EL_ degradation via the proteasome, thereby inhibiting apoptosis. Furthermore, it is worth discussing whether this pathway operates in uninfected bystander cells. ASFV infection at a low MOI (0.1) yielded ~85% bystander PAMs, with only ~15% directly infected (Fig. 5G and H and 6C). Despite this, total Bim protein decreased by >80% (Fig. 1A), suggesting its downregulation occurred in bystander cells. We propose that CD2v, when released from infected cells, mediates this paracrine effect. Supporting this model, purified CD2v alone activated the TPL2–MEK–ERK pathway in naïve PAMs and reduced Bim_EL_ levels by >80% (Fig. 7C), mirroring the effects observed with gradient-purified, heat-inactivated ASFV virions (Fig. 6E) and thereby recapitulating the bystander apoptosis suppression phenotype. These results are consistent with our proposed mechanistic model (Fig. 8), in which CD2v-mediated ERK activation extends anti-apoptotic signaling from infected cells to uninfected bystanders, potentially fostering a pro-survival microenvironment that facilitates viral dissemination. Collectively, our study identified two distinct forms of CD2v-mediated TPL2 activation: one through the soluble protein and another via the virion-incorporated form. We propose that these two forms of CD2v serve complementary biological roles. The soluble form, likely released from infected cells, acts extrinsically as a paracrine modulator to inhibit apoptosis in uninfected bystander cells. Conversely, the virion-associated form functions intrinsically to suppress apoptosis within ASFV-infected cells, thereby securing a favorable intracellular milieu that promotes viral replication from infection onset. This functional duality highlights the multifunctional role of CD2v in ASFV pathogenesis.

Many viruses exploit the ERK1/2 signaling pathway to enhance their replication (45). In our study, while ASFV also activates ERK1/2 to suppress apoptosis, inhibiting this pathway did not significantly affect ASFV replication. Intriguingly, excessive ERK1/2 activation strongly inhibited ASFV replication. This paradox may arise from the pathway’s diverse role in infection: it can promote viral propagation by downregulating Bim_EL_ to inhibit apoptosis, but it can also restrict viral replication by potentiating pro-inflammatory innate immune responses, particularly in macrophages (29). Thus, Selumetinib-mediated inhibition of ERK1/2 may neutralize these opposing effects, resulting in no net change in ASFV replication. Further studies are needed to explore how ERK1/2-mediated non-apoptotic signals influence ASFV replication. As a central hub for extracellular signal transduction, the ERK1/2 pathway is typically activated by virus-receptor interactions involving either viral envelope proteins or infected cell-derived factors (29, 46–48). Using 100 kDa ultrafiltration, we separated virions from sub-100 kDa proteins. Subsequent purification assays and structural protein screening identified CD2v as the key viral factor driving ERK1/2 phosphorylation and Bim_EL_ downregulation. Through the construction of a CD2v-knockout ASFV mutant, we confirmed that CD2v mediates Bim_EL_ degradation and consequently suppresses apoptosis. Notably, sub-100 kDa factors also induced mild ERK1/2 activation, potentially explaining the robust inflammatory cytokine release observed during infection (49).

In summary, we uncover a novel mechanism whereby ASFV CD2v exploits the TPL2-MEK-ERK signaling axis to trigger proteasomal degradation of the pro-apoptotic protein Bim_EL_, thereby suppressing apoptosis in both directly infected and uninfected bystander cells. This mechanism is distinct from other previously reported ASFV anti-apoptotic strategies and is critical for effective viral persistence. Our study broadens the functional understanding of the CD2v protein and uncovers a new dimension of apoptotic regulation by ASFV.

## MATERIALS AND METHODS

### Viruses and cells

The ASFV CADC_HN09 strain (GenBank accession No. MZ614662.1) was provided by the China Animal Disease Control Center (Beijing, China). The WSL cell line, which supports efficient ASFV replication, was established in our laboratory through four rounds of subcloning (20). Both PAMs and WSL cells were cultured in RPMI 1640 medium supplemented with 10% fetal bovine serum, 100 U/mL penicillin, and 100 µg/mL streptomycin at 37°C in a 5% CO₂ atmosphere.

### Antibodies and reagents

The ASFV p30 monoclonal antibody (mAb) was generated in mice, and the recombinant His-tagged extracellular domain (Asp17–Tyr206) of the CD2v protein (ASFV strain HN09) was expressed in HEK-293T cells and purified by Ni-NTA affinity chromatography in our laboratory; both reagents were produced and are maintained in our laboratory (50). β-Actin (66009-1-Ig) mAb was purchased from Proteintech Group, Inc. Rabbit anti-Bim (2933), CASP3 (14220), cCASP3 (9664), p-ERK1/2 (4370), ERK1/2 (4695), and p-Bim (serine 69) (4581) were purchased from Cell Signaling Technology (Boston, MA, USA). Horseradish peroxidase-conjugated goat anti-rabbit (ZB-2301) and anti-mouse (ZB-2305) IgGs were purchased from ZSGB-BIO. DAPI (62248) and Alexa Fluor 488-conjugated goat anti-mouse F(ab′)2 fragment (A11017) were obtained from Thermo Fisher Scientific. The Taq Pro Universal SYBR qPCR Master Mix (Q712-02) and the Annexin V-FITC/PI apoptosis detection kit (A211-01) were purchased from Vazyme Biotech Co., Ltd. Cell counting kit-8 (CCK-8; CK04) was purchased from Dojindo Laboratories. The MagZol Reagent was purchased from Magen Biotech Co., Ltd. The FastKing RT Kit (with gDNase) was purchased from Tiangen Biotech Co., Ltd. Inhibitors, including chlorpromazine (HY-12708), MG132 (HY-13259), CQ (HY-17589A), Selumetinib (HY-50706), Adezmapimod (HY-10256), PMA (HY-18739), and SP600125 (HY-12041) were purchased from Med Chem Express (Monmouth Junction, NJ, USA). Cot inhibitor-2 (SJ-MX5729) was purchased from Shandong Sparkjade Biotechnology Co., Ltd. The dual-luciferase reporter assay system kit (E1980) was purchased from Promega. pAP-1-Luc reporter vector (VT1587) was purchased from YouBio Biotech Co., Ltd.

### Virus infection and titration

To propagate ASFV HN09, PAMs were infected at a multiplicity of infection (MOI) of 0.1 until extensive cytopathic effect was observed. The virus was harvested by three freeze-thaw cycles of infected cells and supernatants. Viral titers were determined by a microtitration infectivity assay using IFA with ASFV p30 mAb (50), and calculated as TCID_50_/mL using the Reed-Muench method. For infection experiments, PAMs or WSL cells were inoculated with ASFV HN09 at an MOI of 0.1 or 0.05 for 2 h at 37°C (5% CO_2_). After adsorption, cells were washed twice with PBS and maintained in fresh medium at 37°C (5% CO_2_) until the designated time points.

### qPCR

Total RNA was extracted from PAMs or WSL cells using MagZol reagent, followed by cDNA synthesis with the FastKing RT Kit (with gDNase), per the manufacturers’ protocols. qPCR was then performed using Taq Pro Universal SYBR qPCR Master Mix, with viral gene expression normalized to β-actin mRNA and quantified via the 2^−ΔΔCt^ method (51). The primers used for qPCR are listed in Table S1.

### Bim knockdown by siRNA

WSL cells were transfected with 40 nM nonspecific siRNA (si*NC*) or Bim-targeting siRNA (si*Bim*-1/2) using Lipofectamine 2000 (Invitrogen, 11668019) at 50%‒70% confluence, following the manufacturer's protocol. Thirty hours post-transfection, cells were infected with ASFV (MOI = 0.1) for 36 h, followed by immunoblotting and flow cytometry analysis. The sequence of siRNA is listed in Table S2.

### Bim-knockout WSL construction

A monoclonal Bim-knockout WSL cell line (WSL-ΔBim) was generated using CRISPR-Cas9 technology as previously described (20). Briefly, parental WSL cells were first transduced with a lentivirus expressing Cas9 and a blasticidin resistance gene (GM-LV3919) to establish a stable line. This Cas9-expressing line was subsequently transduced with a second lentivirus (GM-77116LV) expressing a *Sus scrofa* Bim (NM_001252194.1)-targeting sequence (ACGGAGGCTAAGCGTCGCAA [NC], CGGAAAGGCCTCCTCAGCTC [Bim]) and a puromycin resistance marker. Sequential selection with blasticidin and puromycin yielded a monoclonal population, which was isolated and designated WSL-ΔBim.

### Immunoblotting analysis

Cells were collected by centrifugation (3,000 rpm, 3 min). Both suspended and adherent cells were lysed in RIPA buffer (Beyotime) on ice for 30 min, followed by centrifugation (12,000 rpm, 30 min) to clarify the lysates. Supernatants were mixed with loading buffer, boiled for 10 min, and subjected to immunoblotting analysis (52).

### Flow cytometry analysis

Suspended cells were collected by centrifugation (15 mL tubes), while adherent cells were detached using EDTA-free pancreatic enzymes (37°C, 10 min) and resuspended by gentle pipetting. The cell suspensions were combined and analyzed for apoptosis by flow cytometry following double-staining with Annexin V-FITC/PI (Vazyme detection kit) according to the manufacturer’s protocol.

### Cell viability analysis

PAMs or WSL cells at 50%‒70% confluence in 96-well plates were treated with test compounds at varying concentrations for 24 h (six replicates per concentration), using DMSO-treated cells as negative controls. Cell viability was then assessed using a CCK-8 kit per the manufacturer’s protocol, with 80% viability established as the safety threshold.

### IFA analysis

Cells were fixed with pre-cooled anhydrous ethanol for 15 min, washed twice with PBS, then incubated with anti-p30 mAb (1:1,000 dilution) for 2 h at room temperature. After three PBS washes, samples were incubated with Alexa Fluor 488-conjugated goat anti-mouse F(ab′)₂ secondary antibody for 1 h at room temperature, followed by another three PBS washes. Nuclei were counterstained with DAPI (5 min, room temperature), given two final PBS washes, and imaged by fluorescence microscopy.

### Plasmid construction

Thirty-three ASFV structural protein-coding genes, codon-optimized based on the HN09 strain, were synthesized by Tsingke Biotech (Beijing) and cloned into a C-terminal Myc-tagged pcDNA3.1(+) vector. The Bim_EL_ protein-coding sequence (GenBank no. NM_001252194.1) was cloned into the pcDNA3.1(+) eukaryotic vector using primers listed in Table S1 to generate a recombinant plasmid for expressing Myc-tagged Bim_EL_. All constructs were verified by Sanger sequencing.

### Dual-luciferase reporter assay

WSL cells seeded in 24-well plates were co-transfected with 400 ng of ASFV structural protein plasmid, 400 ng pAP-1-Luc reporter vector, and 8 ng pRL-TK (Promega, E2241) per well using Lipofectamine 2000 for 36 h. Inhibitors or DMSO (control) were added 12 h post-transfection. After removing the medium, cells were washed twice with PBS, and luciferase activity was measured using the dual-luciferase reporter assay system kit following the manufacturer’s protocol.

### Construction of CD2v-knockout ASFV

The donor vector was designed to contain a cassette comprising the EGFP reporter gene under the control of the ASFV p72 promoter. This cassette was flanked by ~1000 bp left and right homologous arms. WSL cells were seeded in 12-well plates and transfected at 50%–70% confluence with 1 μg of the donor vector and 1 μg of EP402R-targeting sgRNA per well for 24 h. Subsequently, the cells and culture medium were harvested, subjected to three freeze-thaw cycles, and then processed through five rounds of limited dilution combined with fluorescent plaque purification on primary PAM monolayers, as previously described (53). Viral DNA from the purified ASFV-ΔCD2v was extracted using the QIAamp DNA Mini Kit (Qiagen, 51304) and further purified with the AMPure XP Purification Kit (Beckman Coulter, A63880). To confirm successful purification of ASFV-ΔCD2v, PCR targeting EP402R and B646L was performed. The primers used for construction and identification of CD2v-knockout ASFV are listed in Table S3.

### Ultrafiltration of viral stocks

A 500 μL aliquot of viral stock was loaded into the upper chamber of a 100 kDa ultrafiltration tube, followed by centrifugation at 3,500 rpm for 10 min. The filtrate (lower chamber fluid), containing proteins <100 kDa, was collected into 1.5 mL microcentrifuge tubes. Approximately 50 μL of the retentate (upper chamber fluid) was resuspended in 450 μL of cell maintenance medium, and the mixture was centrifuged again under the same conditions (3,500 rpm, 10 min). The resulting filtrate (<100 kDa) was collected and resuspended in 450 μL of fresh cell maintenance medium before transfer to 1.5 mL microcentrifuge tubes.

### Purification of virus

To purify the virions, 300 mL of cell-cultured viral suspension was first centrifuged at 3,000 rpm for 30 min to remove cell debris, retaining the supernatant containing the virions. The supernatant was then centrifuged at 8,500 rpm for 6 h, after which the pellet was resuspended in 6 mL of PBS. Next, the Percoll I solution (Percoll 10.8 mL, 10×PBS 1.7 mL, H_2_O 4.5 mL) was layered in an ultracentrifuge tube, and the viral suspension was carefully injected into the bottom. Following centrifugation at 20,000 rpm for 30 min, the virus band (approximately 4 mL) was collected via pipette. The Percoll II solution (Percoll 9 mL, 10× PBS 2 mL, H_2_O 9 mL) was then added to the tube, and the virus suspension was again injected into the bottom. After another round of centrifugation at 20,000 rpm for 30 min, the virus band was collected. Finally, the sample was centrifuged at 33,000 rpm for 1 h, the supernatant was discarded, and the purified virions at the bottom were resuspended in cell maintenance medium.

### Statistical analysis

The data were analyzed using SPSS Statistics v.26.0 (IBM Corp., Armonk, NY, USA). Group differences were assessed with a two-tailed unpaired Student’s *t*-test in GraphPad Prism v.8.0 (La Jolla, CA, USA). Results are presented as mean ± SD from at least three independent experiments, with statistical significance set at *P* < 0.05.

## Data Availability

The data that support the findings of this study are available from the corresponding author upon reasonable request.

## SUPPLEMENTAL MATERIAL

Supplemental material jvi.01952-25-s0001.docx Figures S1 to S5; Tables S1 to S3.

ASM does not own the copyrights to Supplemental Material that may be linked to, or accessed through, an article. The authors have granted ASM a non-exclusive, world-wide license to publish the Supplemental Material files. Please contact the corresponding author directly for reuse.
