# Supplementary material for: The ASFV CD2v protein inhibits apoptosis by inducing proteasomal degradation of BimEL via activation of the TPL2-MEK-ERK signaling pathway
Source: J Virol. 2026 Feb 3;100(3):e01952-25. doi: 10.1128/jvi.01952-25 (PMC13011448; doi:10.1128/jvi.01952-25)
Supplement: Supplemental material — Figures S1 to S5; Tables S1 to S3. [file jvi.01952-25-s0001.docx]

**Supplemental Materials**

**
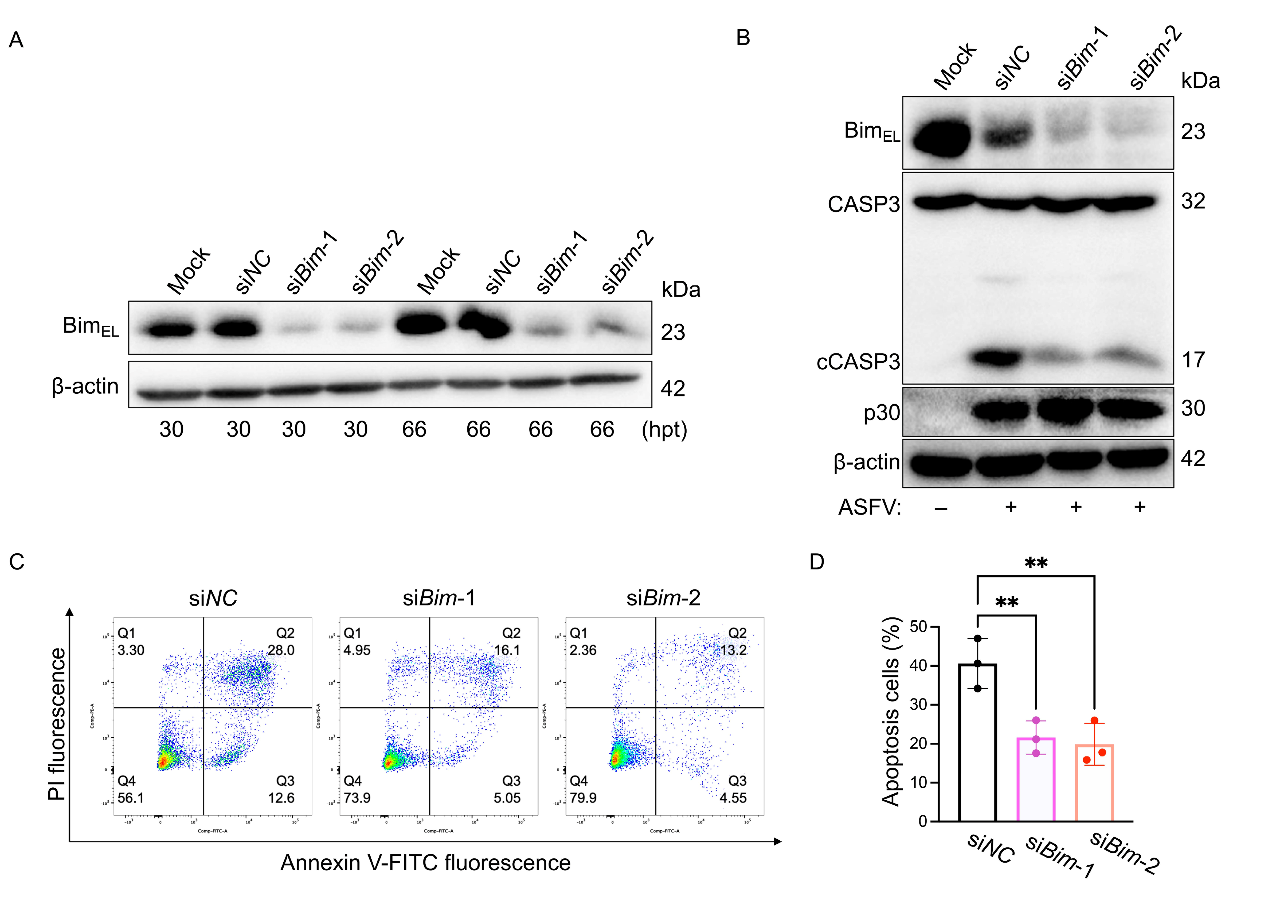
**

**FIG S1** Knockdown of Bim_EL_ suppresses ASFV-induced apoptosis. (A) Immunoblot analysis confirming Bim_EL_ knockdown in WSL cells transfected with non-targeting control siRNA (si*NC*) or two specific siRNAs (si*Bim-1*, si*Bim-2*) at the indicated time points. (B, C) WSL cells were transfected with si*NC*, si*Bim-1*, or si*Bim-2* for 30 h and then infected with ASFV (MOI=0.1) for 36 h. Cells were subsequently analyzed by immunoblotting for caspase-3 (CASP3), cleaved CASP3 (cCASP3), ASFV p30 protein, Bim_EL_, and β-actin (B), or by flow cytometry following Annexin V-FITC/PI staining to quantify apoptosis (C). (D) Quantitative analysis of the flow cytometry data from (C), showing the percentage of Annexin V-FITC-positive (apoptotic) cells. Data are presented as the mean ± SD of three independent experiments (Student's t-test; ***P* < 0.01). hpi, hours post-infection; hpt, hours post-transfection.

**
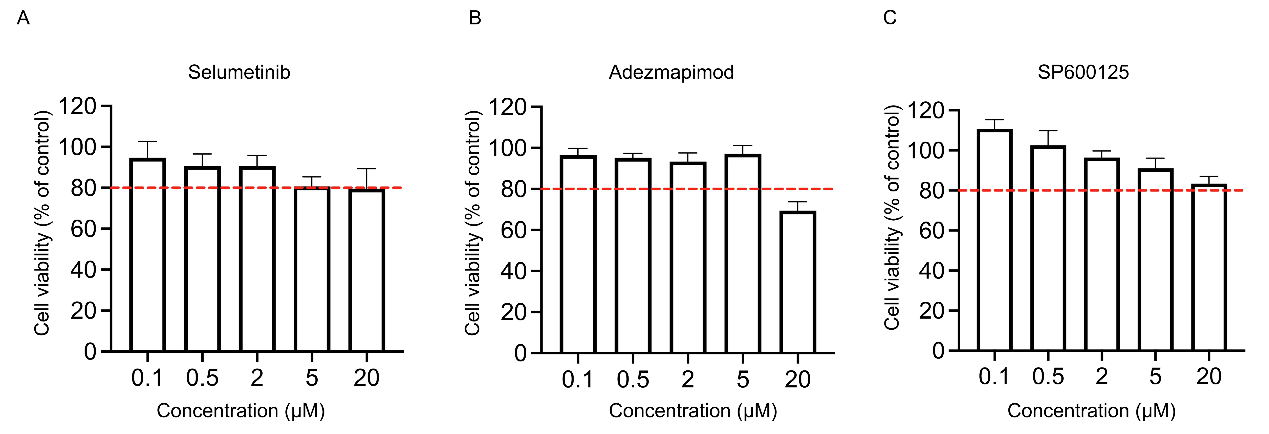
**

**FIG S2** Assessment of non-cytotoxic inhibitor concentrations. (A-C) Cytotoxicity of (A) the MEK inhibitor Selumetinib, (B) the p38 MAPK inhibitor Adezmapimod, and (C) the JNK inhibitor SP600125 in WSL cells was evaluated by CCK-8 assay. The red dotted line denotes the 80% viability threshold.

**
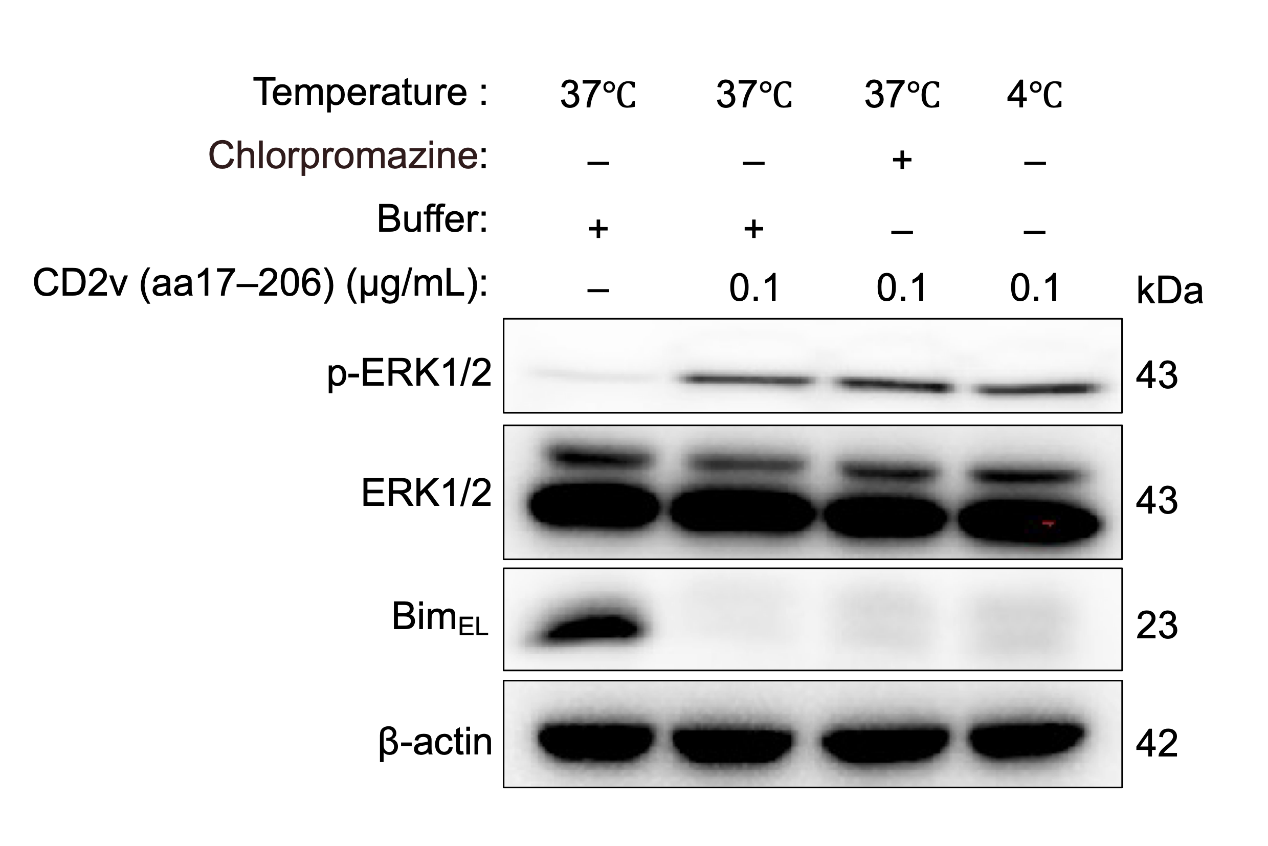
**

**FIG S3** The recombinant ASFV CD2v protein activates ERK1/2 independently of internalization. PAMs were either pre-incubated at 4℃ for 15 min or pretreated with 50 μM chlorpromazine (an endocytosis inhibitor) or DMSO solvent control for 3 h. Cells were then stimulated with purified CD2v protein (Asp17–Tyr206) (0.1 μg/mL) for 30 min at either 4℃ (to block endocytosis) or 37℃. Activation of ERK1/2 and Bim_EL_ downregulation were analyzed by immunoblotting for p-ERK1/2, ERK1/2, Bim_EL_ and β-actin.

**
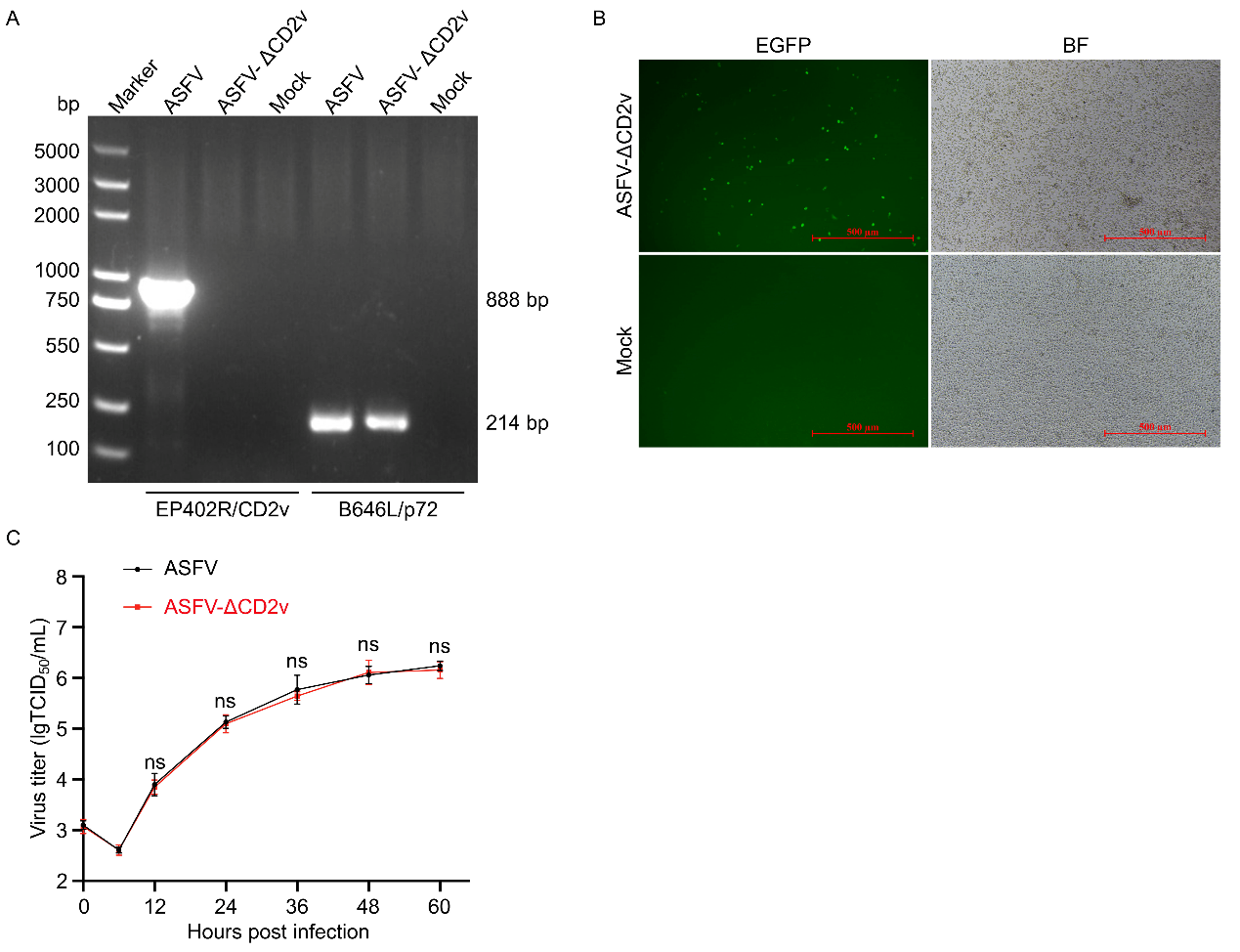
**

**FIG S4** Identification of CD2v-knockout ASFV mutant. (A) PCR identification of the constructed CD2v-knockout ASFV (ASFV-ΔCD2v). Viral DNA was extracted from mock-infected, wild-type ASFV-infected, or ASFV-ΔCD2v-infected PAMs (MOI=0.1, 48 h) and amplified using primers targeting *B646L* (p72) and *EP402R* (CD2v) genes (Table S3). (B) Fluorescence microscopy validating EGFP expression in PAMs infected with ASFV-ΔCD2v (MOI=0.05, 24 h). BF, Bright field. (C) Growth kinetics of wild-type ASFV and ASFV-ΔCD2v in PAMs. Cells were infected (MOI=0.1), harvested at the indicated time points, and viral titers were determined by TCID_50_ assay. Data are represented as the mean ± SD of three independent experiments (Student's t-test; ns, no significance).


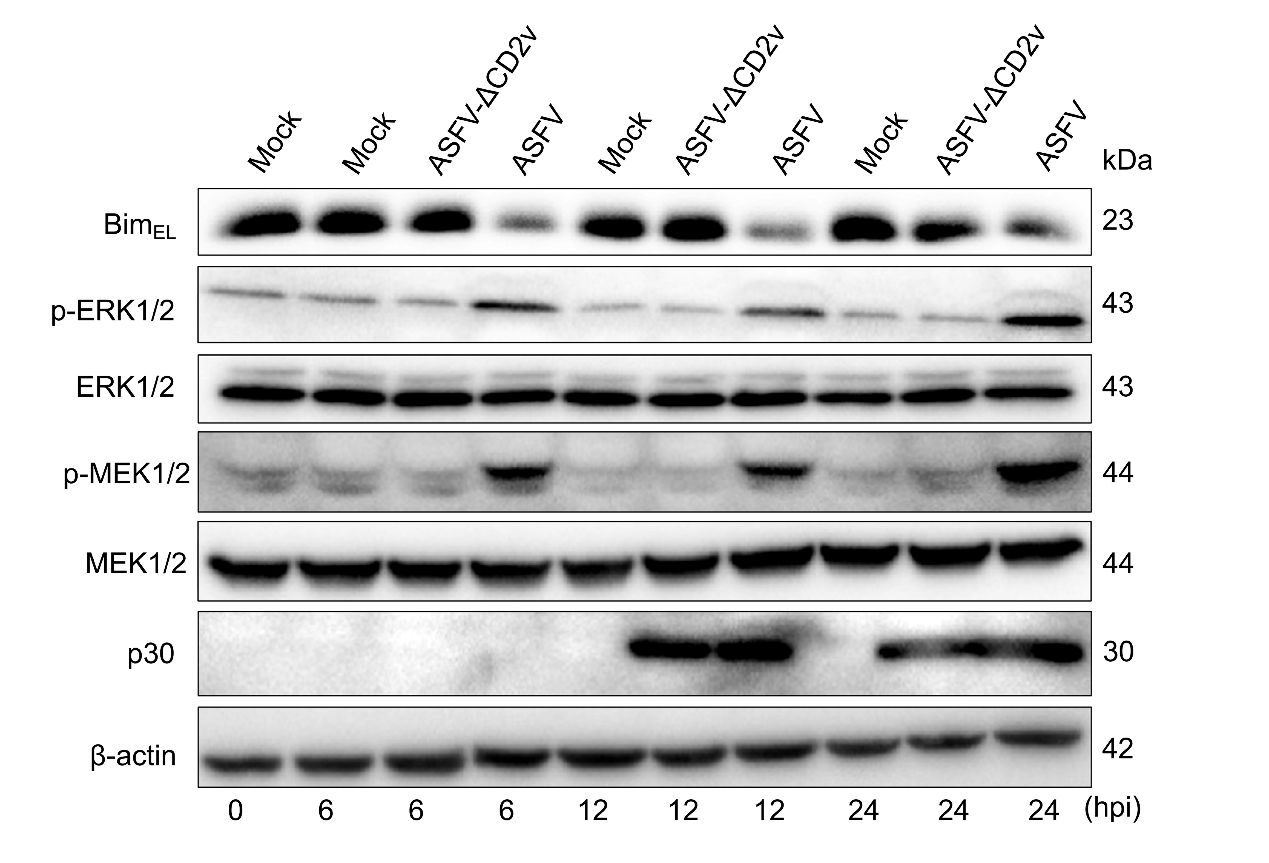


**FIG S5** ASFV CD2v activates ERK1/2 to drive Bim_EL_ degradation in WSL cells. WSL cells were mock-infected or infected with wild-type ASFV or ASFV-ΔCD2v (MOI=0.1) for the indicated time points. Cell lysates were subjected to immunoblot analysis for p-ERK1/2, ERK1/2, p-MEK1/2, MEK1/2, Bim_EL_, ASFV p30, and β-actin.

**TABLE S1** Primers for *Bim* mRNA quantification and Bim_EL_ plasmid construction

| Name | Sequence (5′-3′) | Application |
| --- | --- | --- |
| pBim-F | GGTAATCCGGAAGGAGAAGGG | Quantification of *Bim* mRNA by quantitative real-time PCR |
| pBim-R | ACGAAGATGAAAAGCGGGGA |  |
| pβ-actin-F | CTCCATCATGAAGTGCGACGT |  |
| pβ-actin-R | GTGATCTCCTTCTGCATCCTGTC |  |
| Bim-Myc-F | TGGTACCGAGCTCGGATCCGCCACCATGGCAAAGCAACCTTCCGATGTAAGTTCT | Construction of a Myc-tagged Bim_EL_ expression plasmid |
| Bim-Myc-F | TGGATATCTGCAGAATTCTTACAGATCCTCTTCAGAGATGAGTTTCTGCTCAATGTAAGGGGGAGGGAGGGTGTGAG |  |

**TABLE S2** The designed siRNAs for Bim knockdown in WSL cells

| Name | Sequence (5′-3′) | |
| --- | --- | --- |
|  | sense | antisense |
| si*Bim*-1 | GAGACGAAUUUAAUGCAUATT | UAUGCAUUAAAUUCGUCUCTT |
| si*Bim*-2 | CGAUGUAAGUUCUGAGUGUTT | ACACUCAGAACUUACAUCGTT |
| si*NC* | UUCUCCGAACGUGUCACGUTT | ACGUGACACGUUCGGAGAATT |

**TABLE S3** Primers for construction and identification of CD2v-knockout ASFV mutant

| Name | Sequence (5′-3′) |
| --- | --- |
| sgRNA-sense-F | CACCGTTGGGTAGTAGCGGGATACT |
| sgRNA-sense-R | AAACAGTATCCCGCTACTACCCAAC |
| Left-arm-F | GTACCGGGCCCCCCCTCGAGCTCCATTCTCATTGCATGCTTG |
| Left-arm-R | CTTTTCCTCCGGCGACCCTTTTTATGAACATATGTTTTATAATATAGTATCAAAAAC |
| p72-promoter-EGFP-F | AAGGGTCGCCGGAGGAAAAG |
| p72-promoter-EGFP-R | TTACTTGTACAGCTCGTCCATGCCG |
| Right-arm-F | GGACGAGCTGTACAAGTAATATGTACTATATATTAATTATTTAACCTTTCAAG |
| Right-arm-R | ATCCCCCGGGCTGCAGGAATTCCGCTTCGGCTCGCTTCAT |
| B646L-F | TATCGGTGGAGGGAACCAGT |
| B646L-R | TACGTTGCGTCCGTGATAGG |
| CD2v-F | CACTAGCTACATGTGGAAAAGCAGG |
| CD2v-R | GGGTAGATAATGGCGGGATATTG |

F: forward primer; R: reverse primer.
